# Combinatorial Screening for Transgenic Yeasts with High Cellulase Activities in Combination with a Tunable Expression System

**DOI:** 10.1371/journal.pone.0144870

**Published:** 2015-12-21

**Authors:** Yoichiro Ito, Mamoru Yamanishi, Akinori Ikeuchi, Chie Imamura, Takashi Matsuyama

**Affiliations:** 1 Matsuyama Research Group, TOYOTA Central Research and Development Laboratories Incorporation, Nagakute, Aichi, Japan; 2 Biotechnology Laboratory, TOYOTA Central Research and Development Laboratories Incorporation, Nagakute, Aichi, Japan; Virginia Tech, UNITED STATES

## Abstract

Combinatorial screening used together with a broad library of gene expression cassettes is expected to produce a powerful tool for the optimization of the simultaneous expression of multiple enzymes. Recently, we proposed a highly tunable protein expression system that utilized multiple genome-integrated target genes to fine-tune enzyme expression in yeast cells. This tunable system included a library of expression cassettes each composed of three gene-expression control elements that in different combinations produced a wide range of protein expression levels. In this study, four gene expression cassettes with graded protein expression levels were applied to the expression of three cellulases: cellobiohydrolase 1, cellobiohydrolase 2, and endoglucanase 2. After combinatorial screening for transgenic yeasts simultaneously secreting these three cellulases, we obtained strains with higher cellulase expressions than a strain harboring three cellulase-expression constructs within one high-performance gene expression cassette. These results show that our method will be of broad use throughout the field of metabolic engineering.

## Introduction

*Saccharomyces cerevisiae* is a well-established metabolic engineering platform that is used in the production of many valuable industrial compounds [1–4]. To increase the yields of these industrial compounds, metabolic flux must be maximized and the accumulation of by-products and intermediates must be minimized [5]. Therefore, the ability to simultaneously coordinate the activities of many different enzymes, including those that are heterogeneous, is essential [6–8]. Traditional approaches for maximizing metabolic flux include over-expressing key enzymes by using high-performance promoters [9–11], increasing the copy number of the target gene [12], and improving catalytic activity by means of directed evolution [13, 14]. Recently, combinatorial screening methods have been developed to aid in maximizing metabolic flux [7, 8, 12, 15–18]; one such example of their use has been the fine-tuning of protein expression to balance the expression of cellulase on the yeast cell surface [12].

Consolidated bioprocessing is a cost-effective process to convert lignocelluloses into desired products in a single step without the need for additional enzymes [19–21]. By using *S*. *cerevisiae* in recombinant consolidated bioprocessing strategies, a number of studies have improved cellulase activity by selecting highly secreted cellulases [22] or by using cellulases and cellulosome systems of complexed cellulases bound to the cell surface [23–25]. However, cellulase-expressing yeast strains capable of assimilating cellulose at an industrial level are yet to be produced with stable genome-integrated constructs and not plasmids. Therefore, genome-recombinant yeast strains with higher levels of secretory cellulase expression are needed for the further development of consolidated bioprocessing.

Recently, we constructed a highly tunable protein expression system that enabled the simultaneous, graded expression of multiple proteins [26]. By using different combinations of three gene-expression control elements—number of transcriptional activator binding sites, type of core promoter, and type of terminator region—our system produced a yeast strain with 8-fold greater green fluorescent protein (GFP) expression than a strain containing a standard construct harboring the *TDH3* promoter and *CYC1* terminator. Furthermore, our system showed a broad GFP-expression dynamic range of 30,000. Comparable results were obtained for the expression of secretory cellulases.

In the present study, we conducted combinatorial screening for transgenic yeasts secreting high levels of cellulases for the fermentation of ethanol directly from crystalline cellulose by using a set of gene expression cassettes that were constructed in our previous study [26]. The cellulase-expressing yeasts obtained from the combinatorial screening produced higher yields of ethanol than the strains used in previous reports [22, 26].

## Materials and Methods

### Host strain and media

*Escherichia coli* JM109 was used as the host cell for DNA manipulation. Luria-Bertani (LB) medium supplemented with 100 mg/l ampicillin was used for *E*. *coli* cell culture to select the transformants. *S*. *cerevisiae* strain W303-1A (*MATa leu2-3*,*112 trp1-1 can1-100 ura3-1 ade2-1 his3-11*,*15*) was used as the host strain for genetic manipulations. Yeast transformants were cultured in synthetic complete medium (SD) or SD-derived selection medium (SD-LEU, SD-URA, or SD-TRP) containing 0.67% Yeast Nitrogen Base without amino acids (YNB) (Difco, Detroit, MI); 0.082% Complete Supplement Mixture (CSM) without uracil, leucine, or tryptophan (ForMedium, Norfolk, UK); adenine (40 mg/L); and 2% glucose. SD-derived pre-culture media for the cellulase assay contained 0.67% YNB, 0.082% CSM-URA-LEU-TRP, adenine (40 mg/L), 1% (w/v) casamino acids, and 2% glucose.

### Construction of plasmids

Secretory expression constructs of cellulases were used as described in our previous paper [26]. Briefly, genes encoding cellobiohydrolase 1 (CBH1) [22], which is derived from *Talaromyces emersonii* (GenBank accession no. AAL89553); a thermostable mutant of cellobiohydrolase 2 (CBH2) derived from *Phanerochaete chrysosporium* [14]; and endoglucanase 2 (EG2), which is derived from *Trichoderma reesei* (GenBank accession no. AAA34213) [27] were used in this study. Secretory signal peptide of the glucoamylase gene from *Rhizopus oryzae* (DDBJ accession no., 304862) was added to the 5′ end of each cellulase gene as described in a previous study [26].

All genetic constructs were cloned into the pSP73 vector (Promega, Madison, WI) by means of standard restriction enzyme digestion and ligation techniques or by using an In-Fusion Advantage PCR cloning kit (Clontech, Mountain View, CA).

### Combinatorial screening

The expression cassettes in Table 1 for the cellulase genes were inserted by means of single-crossover integration [2, 28]. A mixture (total volume, 20 μg) comprising 5 μg of DNA fragments of the cellulase-expressing constructs within the L5TR, L5TC, L1TC, and L1CC cassettes was used for the transformations to construct a library of gene expression cassettes with various cellulase expression levels. Before transformation, plasmids carrying the mixture of these four constructs were digested with the corresponding restriction enzyme (*Cla*I for *LEU2*; *Nco*I for *URA3*; and *Hind*III for *TRP1*). Yeast transformation was performed as described previously [29]. Briefly, yeast cells were grown overnight in yeast extract–peptone–dextrose (YPD) medium, then diluted ten-fold with YPD and incubated for 5 h. Cells were precipitated and washed with deionized water, and then mixed with transformation solution (120 μl of 60% PEG3350, 5 μl of 4 M lithium acetate, 10 μl of 1 M DTT, and 10 μl of 10 mg/ml denatured salmon sperm DNA) and plasmid DNA. The mixture was incubated at 42°C for 40 min, and then spread on suitable selection media. This transformation procedure was repeated in the order *CBH2*, *EG2*, *CBH1*. In each transformation cycle, several hundred colonies were collected and cultured as hosts for the next round of cellulase cassette introductions to obtain transformants that ideally had one of a possible 64 random insertions. A total of 368 individual transformants were produced and cellulase activity was measured by using deep 96-well plates and 2% (w/v) Avicel buffered in 50 mM acetate buffer (pH 5.0) as the substrate, as described below. The five transformants with the highest cellulase activity were identified and cellulase activity, the copy number of each genome-integrated cellulase gene, and the type of cellulase construct inserted into the yeast genome were assessed by using diagnostic polymerase chain reaction (PCR).

**Table 1:** List of 12 expression cassettes used in the present study for the combinatorial screening.

Name	Cellulase gene	Number of LNV1 binding sites	Core promoter	Terminator	Marker
L5TR_CBH1	CBH1	5	CP_TDH3_	*RPL41Bt*	*LEU2*
L5TC_CBH1	CBH1	5	CP_TDH3_	*CYC1t*	*LEU2*
L1TC_CBH1	CBH1	1	CP_TDH3_	*CYC1t*	*LEU2*
L1CC_CBH1	CBH1	1	CP_CYC1_	*CYC1t*	*LEU2*
L5TR_CBH2	CBH2	5	CP_TDH3_	*RPL41Bt*	*URA3*
L5TC_CBH2	CBH2	5	CP_TDH3_	*CYC1t*	*URA3*
L1TC_CBH2	CBH2	1	CP_TDH3_	*CYC1t*	*URA3*
L1CC_CBH2	CBH2	1	CP_CYC1_	*CYC1t*	*URA3*
L5TR_EG2	EG2	5	CP_TDH3_	*RPL41Bt*	*TRP1*
L5TC_EG2	EG2	5	CP_TDH3_	*CYC1t*	*TRP1*
L1TC_EG2	EG2	1	CP_TDH3_	*CYC1t*	*TRP1*
L1CC_EG2	EG2	1	CP_CYC1_	*CYC1t*	*TRP1*

### Secreted cellulase activity

Cellulase activity was measured as described previously with slight modification [14, 26]. Briefly, colonies of cellulase-expressing transformants were transferred into 500 μl of SD medium without leucine, tryptophan, and uracil supplemented with 1% casamino acids in a deep 96-well plate and grown at 30°C with shaking at 1800 rpm for 24 h in a specialized shaker for deep-well plates (MBR-022UP; Taitec, Aichi, Japan). Next, a 100-μl aliquot of each culture was inoculated into 500 μl of SD medium without leucine, tryptophan, and uracil supplemented with 1% casamino acids and again grown at 30°C with shaking at 1800 rpm for 24 h. After centrifugation at 3000 rpm for 10 min, the cellulase activity of 5-μL aliquots of supernatant was determined by measuring the concentration of reducing sugar with 2% (w/v) Avicel buffered in 50 mM acetate buffer (pH 5.0) as the substrate, as described previously.[14, 26] Cellulase reactions were conducted at 50°C for 4 h. All measurements were performed in triplicate.

### Diagnostic PCR

The genomes of the cellulase-expressing transformants were isolated after overnight culture by using a Dr. GenTLE (from Yeast) High Recovery kit (TakaraBio, Shiga, Japan). Diagnostic PCR was performed by using the extracted yeast genomes of the variants as the template and two primer sets, one annealing to the end of the 5′-recombination site and the beginning of the secretory signal peptide, and the other annealing to the end of the cellulase gene and the beginning of the marker genes. The PCR primer sequences used are as follows: 5′-AATTCGCACGTAGACTGGCTTGAA-3′ for D1, 5′-GCATGCAGCAGCAGAAACCAACAAAG-3′ for D2, 5′-GACTTCTCTCAACACGGTGGTTTG-3′ for D3, 5′-TGAACACACATGAACAAGGAAGTAC-3′ for D4, 5′-GATCTGTATCTGCACCTAGATCGAA-3′ for D5, 5′-CCATTGCTTCAACAAGCTGGTTG-3′ for D6, 5′-CTGTTCGGAGATTACCGAATCGGAT-3′ for D7, 5′-GCATATTTATTTACATTTTGTCGGAATGAA-3′ for D8, 5′-CAATAGACAAGCGATTTTAACAGA-3′ for D9 and 5′-CCTATATTATATATATAGTAATGTCGTTGGA-3′ for D10. The cellulase construct was determined by comparing the lengths of the PCR products with those of the control plasmid for the L5TR, L1TC, or L1CC expression cassettes.

### Determination of gene copy number

To quantify the number of genes responsible for the target cellulases, real-time PCR with SYBR Green I was performed by using a SYBR Premix Ex-Taq II kit (TakaraBio) and an ABI PRISM 7000 Sequence Detection System (Life Technology, Carlsbad, CA). The *UBC6* gene was used as the internal standard. The PCR primers used are as follows: 5′-CGGCAAATACAGGTGATGAAAC-3′ and 5′-TCCTCCAACGAGATGACTTTTTC-3′ for the *UBC6* gene, 5′-AAAACGGTGCTGTCGTCTTG-3′ and 5′-TCCAAGGCACAGTTTTGAGC-3′ for the *CBH1* gene, 5′-GCTCATCCGTTTCTTCGGTATC-3′ and 5′-GGATTGTTAGCAGATGGTGGTG-3′ for the *CBH2* gene, and 5′-TTGGGAGCCTACTGTATTGTCG-3′ and 5′-TGGAAGCCAGTTGAGACCATAG-3′ for the *EG2* gene. Thermocycling conditions consisted of 40 cycles of 10 sec at 94°C and 1 min at 60°C, followed by a dissociation step. The threshold value for determining the threshold cycle number was set manually. The copy number of the cellulase gene was normalized to that of the *UBC6* gene. Measurements were performed in duplicate.

### SDS-PAGE of culture supernatants

To identify which protein band on SDS-PAGE corresponded with which cellulase, we constructed four control transgenic yeast strains: three strains each expressing one of the cellulases and harboring the L5TR cassette, and one strain simultaneously expressing all three cellulases and harboring the *TDH3* promoter and the *CYC1* terminator. After cultivation for 72 h, the culture supernatants were collected, passed through a 0.45-μm hydrophilic filter, and concentrated 100-times by using an ultrafiltration device (Vivaspin 30K, GE Healthcare). Immediately before use, the concentrates were diluted 10-times and aliquots (10 μl) were used for SDS-PAGE. Following CBB staining, the gel was scanned with an image scanner (ImageScanner III, GE Healthcare) and the image was analyzed by using the ImageJ software [30].

### Activities of secreted cellulases

To separately determine the activities of the secreted CBH1 and EG2, we used the cellulase specific substrates resorufin-labeled cellobiose and azurine-crosslinked hydroxyethylcellulose (AZCL-HE-cellulose; Megazyme International Ireland Ltd., Wicklow, IRL), respectively. First, we assayed the activity of CBH1 secreted into the supernatant by means of SDS-PAGE analysis with resorufin-labeled cellobiose using a MarkerGene^−^ Fluorescent Cellulase Assay Kit (Marker Gene Technologies, Inc., OR; [31]). Briefly, 0.25 mM substrate was added to 10-times diluted supernatant and the mixture was incubated for 30 min at room temperature. After the addition of stop buffer, fluorescence intensity was measured (excitation 535/25 nm and emission 590/20 nm) by using an Infinite F500 microplate reader (Tecan Group Ltd., Mannedorf, CHE).

Next, we assayed the activity of EG2 secreted into the supernatant by using the substrate AZCL-HE-cellulose. Diluted supernatants were added to 50 mM citric acid buffer (pH 5.0) containing 200 mg/l AZCL-HE-cellulose and the mixture was incubated for 30 min at 40°C. After the addition of 2% w/v Trizma base solution (Sigma-Aldrich, St. Louis, MO, USA) to stop the reaction, the absorbance of the filtrate at 590 nm was measured against a blank with a SpectraMax Plus 384 microplate reader (Molecular Devices LLC., CA).

### Fermentation of Avicel to ethanol

Ethanol fermentation was investigated as described previously [26]. A standard strain (SW), the L5TR strain (HR), the three strains obtained from the combinatorial screening, and a reference strain expressing no cellulase were cultured in 50 ml of YPD medium supplemented with 40 mg/l of adenine in baffled, 200-ml shake flasks incubated at 30°C on a rotary shaker at 130 rpm for 3 days. Seventeen milliliters of each culture was then added to a 10-ml shake flask containing 0.38 g of Avicel PH-105 (Sigma-Aldrich, St Louis, MO), 1.9 ml of 0.5 M citrate buffer (pH 5.0), and 100 μl of β-glucosidase (Novozyme 188, Sigma-Aldrich) (final Avicel concentration, 20 g/l). The flask was sealed with a rubber bung and a check valve to maintain anaerobic conditions and stirred for 7 days. Samples were taken on days 0, 1, 2, 3, 5, and 7 and the glucose and ethanol content was analyzed with a high-performance liquid chromatograph (Shimadzu, Kyoto, Japan) containing a Biorad HPX 87H column (Biorad, Hercules, CA). The column was eluted at 60°C with 0.5 g/l H_2_SO_4_ at a flow rate of 0.8 ml/min. A refractive index detector (model RID-10A, Shimadzu) was used for detection.

## Results and Discussion

### Preparation of cellulase-expressing constructs for combinatorial screening

**Fig 1:**
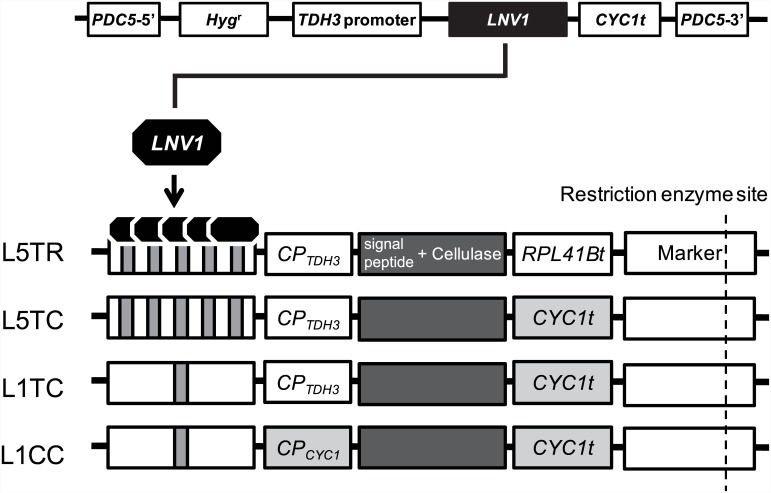
Scheme of the genome-integrated multiple protein-expression system. LNV1 expression construct (upper) and the four protein-expression cassettes (lower) used in this study. LNV1 was the transcription activator used in this study. The LNV1 expression construct was integrated into the *PDC5* locus on the yeast genome. The protein expression level of yeast strains carrying each of the cassettes was regulated by three factors: number of LNV1 binding sites, type of core promoter, and type of terminator region. Each cassette was introduced by means of single-crossover integration by using the restriction enzyme site on the marker gene. In the figure, the protein expression cassettes are shown in decreasing order of the level of expression conferred from top to bottom.

The tunable protein-expression system used in this study was composed of one construct (LNV1) expressing an artificial transcriptional activator, and expression cassettes containing the target transgenes (Fig 1). LNV1 was composed of three well-characterized components: the DNA binding site from the lexA protein [32], a nuclear transfer signal [33], and the transcriptional activation domain VP16 [34]. An LNV1 genome-integration cassette was introduced into the yeast strains at the *PDC5* locus, the deletion of which does not change the phenotype or pyruvate decarboxylase activity [35, 36]. The transgene expression level is dependent on the combination of the number of LNV1 binding sites, the type of core promoter (CP), and the type of terminator region used in the expression cassette. The expression cassettes used in this study were named LxAB, where x is the number of LNV1 binding sites, A is the type of core promoter (“T” or “C” indicating CP_TDH3_ or CP_CYC1_, respectively), and B is the initial letter of the terminator region (“R” or “C” indicating the *RPL41B* terminator [*RPL41Bt*] or the *CYC1* terminator [*CYC1t*], respectively). *RPL41Bt* had the highest 3′-UTR activity in a previous study [37] and the *CYC1* terminator is a commonly used terminator [38, 39]. In the present study, we used four expression cassettes (L5TR, L5TC, L1TC, and L1CC) to express three cellulase genes (*CBH1*, *CBH2*, and *EG2*). L5TR, L5TC, L1TC, and L1CC showed expressions of EG2 of 4.1, 2.2, 1.0, and 0.5, respectively, which was comparable to that of a standard strain containing the *TDH3* promoter and the *CYC1* terminator [26].

### Combinatorial screening to optimize the production of secretory cellulases

**Fig 2:**
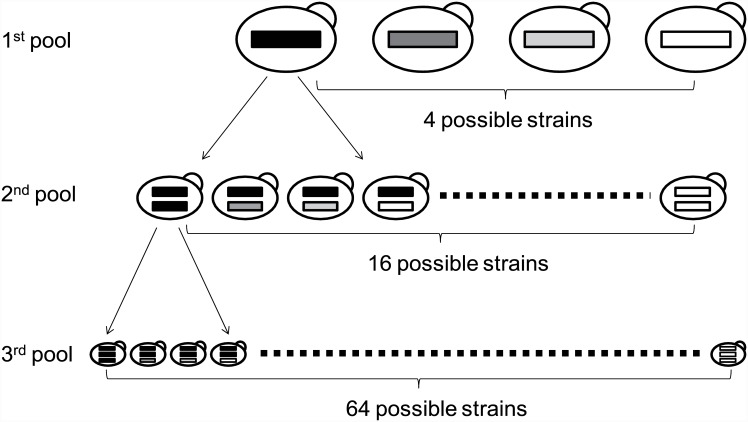
Scheme of the combinatorial screening. Preparation of the 64 (4 × 4 × 4) possible strains. Transformation of cellulase cassettes was performed repeatedly in the order CBH2, EG2, CBH1. Screening of the cellulase activity of the 64 possible strains was also performed.

Combinatorial screening was conducted to optimize the secretion level of the three cellulases (*CBH1*, *CBH2*, and *EG2*) for the effective digestion of the crystalline cellulose Avicel (Fig 2). The mixture of the four constructs containing a cellulase gene was integrated into the yeast genome, which contained an LNV1-expressing construct. Hundreds of transformant colonies were collected and mixed; therefore, the pool was large enough to include transgenic yeasts harboring a cellulase construct with each of the four gene cassettes. This procedure was repeated in the order *CBH2*, *EG2*, *CBH1*. After three rounds of transformation, we obtained a pool of 64 possible transgenic yeasts each simultaneously expressing three cellulase genes.

**Fig 3:**
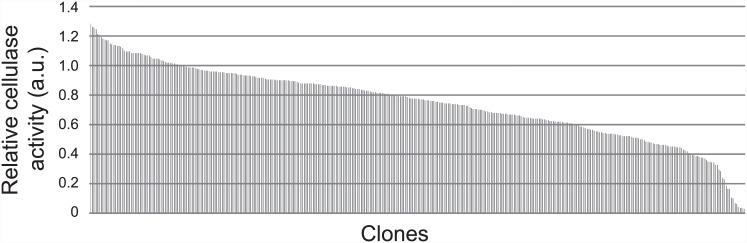
Relative cellulase activities of 368 combinatorially prepared transformants. Relative cellulase activity was normalized to the cellulase activity of the HR strain with Avicel cellulose as the substrate. Approximately 15% of the variants had higher cellulase activity than that of the HR strain.

To investigate which combination of cellulase and gene cassette had the highest cellulase activity, the cellulase activity of the supernatants from 368 transformants from the transformant pool was measured with Avicel cellulose as the substrate (Fig 3). In our previous study, the strain simultaneously expressing *CBH1*, *CBH2*, and *EG2* with the L5TR cassette (HR strain) had 1.6-fold greater cellulase activity than that of the standard strain expressing the three cellulase genes under the *TDH3* promoter and the *CYC1* terminator (SW strain) [26]. In the present study, the transformants showed a range of cellulase activities from 1.2 to 0.02 relative to the HR strain, and approximately 15% of the transformants had a higher cellulase activity than that of the HR strain (Fig 3).

**Fig 4:**
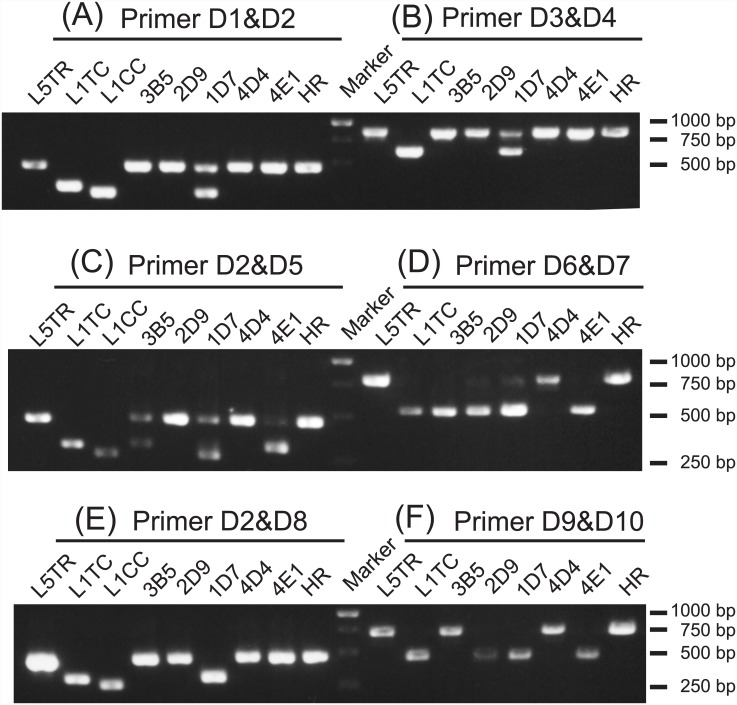
Diagnostic polymerase chain reaction (PCR) assay of the variants obtained from the combinatorial screening. PCR was performed with the indicated primer sets in Materials and methods. The type of genome-integrated cellulase construct was determined from the lengths of the PCR products. PCR products of the transformants carrying the CBH1 (A, B), CBH2 (C, D), and EG2 constructs (E, F). The PCR products for the core promoter (A, C, E) and terminator regions (B, D, F) were amplified.

The five transformants with the greatest cellulase activity were investigated in detail (Table 2). The cellulase activity of these five transformants was approximately 1.2-fold greater than that of the HR strain (P < 0.05). To determine which expression cassette the transformants contained, diagnostic PCR was conducted with a set of specific primers (Fig 4 and summary in Table 2). All five strains contained a construct expressing *CBH1* under the L5TR cassette, which was the cassette that conferred the highest protein activity [26]. For the expression of *CBH2* and *EG2*, the L5TR or L5TC cassette was preferentially selected. These results were consistent with previous results in that the activity of the transformant increased with the increasing performance of the expression cassette it contained [26].

**Table 2:** Combinatorial screening of cellulase constructs.

Transformant	Relative activity of secreted cellulases (a.u.)^1^	Genome integrated construct^2^ (Gene copy number^3^)			Ethanol production (g/l)^4^
		CBH1	CBH2	EG2	
3B5	1.22 ± 0.08	L5TR (2.6)	L5TC/L1TC (3.1)	L5TR (0.9)	5.2 ± 0.4
2D9	1.19 ± 0.08	L5TR (4.2)	L1TC (3.3)	L5TC (1.0)	5.7 ± 0.2
1D7	1.18 ± 0.08	L5TR/ L1CC (2.7)	L5TC/L1CC (3.7)	L1TC (1.3)	n.d.
4D4	1.17 ± 0.08	L5TR (1.9)	L5TR (1.6)	L5TR (0.9)	4.8 ± 0.3
4E1	1.12 ± 0.08	L5TR (2.4)	L5TC/L1TC (2.8)	L5TC (0.9)	n.d.
HR strain	1.00 ± 0.08	L5TR (1.0)	L5TR (0.8)	L5TR (0.9)	3.8 ± 0.3

^1^An aliquot of supernatant was incubated at 50°C for 4 h in the presence of 2% Avicel. Cellulase activity was calculated from the amount of reducing sugar present in the supernatant after incubation. Values shown are normalized to that of the transformant carrying only L5TR expression cassettes. Measurements were performed in triplicate.

^2^Determined as the length of the diagnostic polymerase chain reaction (PCR) products (see Fig 4).

^3^Determined from the quantification of the cellulase genes in the yeast genome by means of quantitative PCR. Average values from two independent experiments are shown.

^4^Ethanol concentrations at 168 h of fermentation with the external addition of β-glucosidase (see Fig 4B).

n.d., not determined

In strains 3B5, 1D7, and 4E1, multiple DNA fragments were detected (Fig 4). This result can be explained by the fact that single-crossover transformation frequently results in the integration of multiple gene copies at the target locus. We determined the copy number of each construct in the five transformants by means of quantitative PCR because the copy number of the genome-integrated transgenes directly influences expression level [2]. All five strains contained multiple copies of the *CBH1*- or *CBH2*-expressing constructs; however, each strain contained only a single copy of the *EG2*-expressing construct (Table 2). Because the composition of the cellulase expression cassettes was the same in strains 4D4 and HR, the difference in the cellulase activities of these strains was considered to be dependent upon the copy number of *CBH1* and *CBH2*, but not that of *EG2* (Table 2).

**Fig 5:**
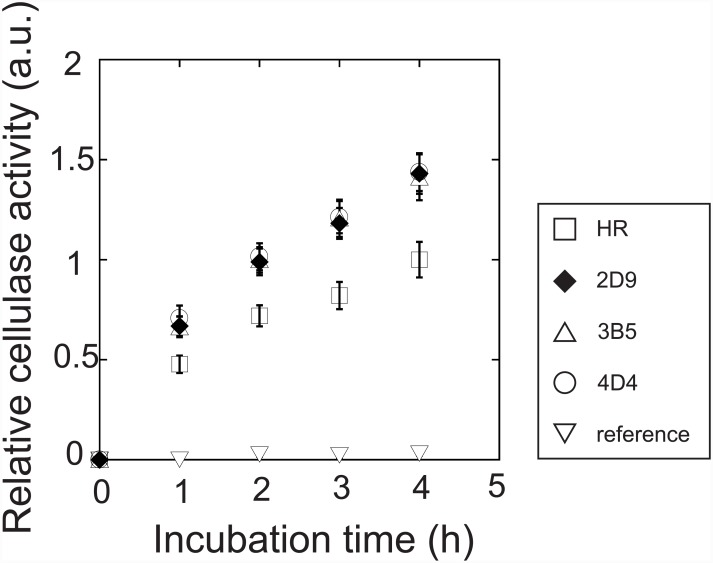
Cellulase activity of the transgenic strains obtained from the combinatorial screening. Squares, diamonds, triangles, circles, and inverted triangles represent the HR, 2D9, 3B5, 4D4, and reference strains, respectively. Cellulase secretion was assessed by culturing the cells in yeast extract–peptone–dextrose medium and then measuring the cellulase activity by using Avicel cellulose as the substrate.

To verify the result of the cellulase activity from the combinatorial screening, the three strains with the highest activity—3B5, 2D9, and 4D4 (Table 2)—were investigated by means of the procedure we used in our previous study [38]. After each strain was incubated for 3 days in YPD medium, the supernatants were sampled and cellulase activity was assessed every hour for four hours with Avicel cellulose as the substrate. From 1 to 4 h after incubation, the activities of all the strains showed linearity, and the cellulase activity of the three strains were equal and approximately 1.4-fold greater than that of the HR strain (Fig 5). The difference in the relative activities compared with those from the combinatorial screening and in the measurement of their kinetics (1.2- vs. 1.4-fold greater) is likely due to the type of medium used or duration of incubation; therefore, these results were considered to be consistent with those obtained from the combinatorial screening.

### Amounts of secreted cellulases

**Fig 6:**
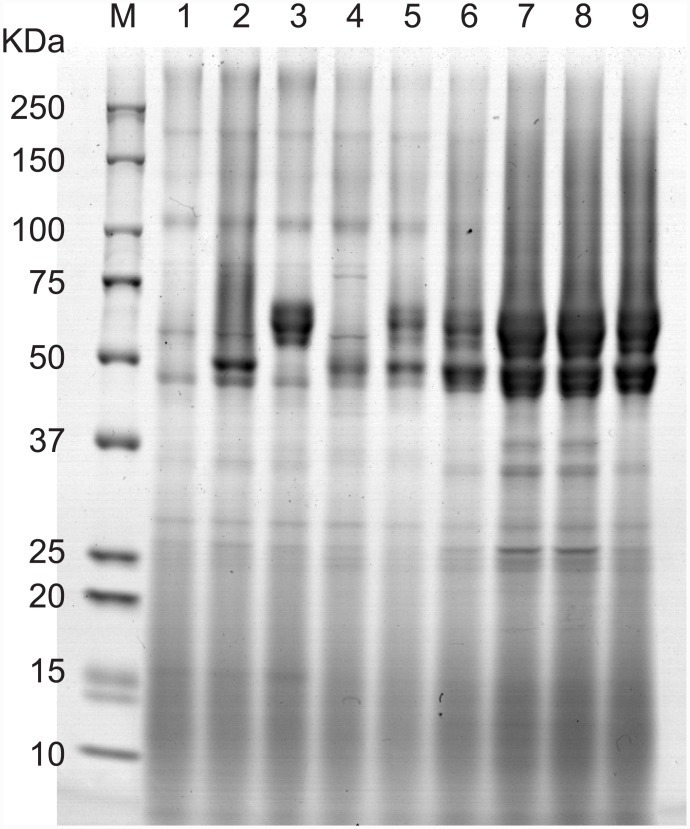
SDS-PAGE analysis of secreted cellulases. Lane 1, SW strain; Lane 2, CBH1 strain; Lane 3, CBH2 strain; Lane 4, EG2 strain; Lane 5, CBH1 + CBH2 + CBH3 with *TDH3pro* + *CYC1t* strain; Lane 6, HR strain; Lane 7, 3B5 strain; Lane 8, 2D9 strain; Lane 9, 4D4 strain.

To help elucidate the relationship between the total activity of the cellulases produced by each strain and the corresponding genotype of the cellulase transgenes, the amounts of cellulases secreted into the culture supernatant were determined by means of SDS-PAGE (Fig 6). Glycosylation during the process of secretion has been shown to increase the molecular weight of cellulases secreted by *S*. *cerevisiae* [40]; therefore, although the molecular weights of the cellulases used in the current study should be CBH1, 49 KDa; CBH2, 46 KDa; and EG2, 44 KDa; in our previous studies, CBH2 has been observed on SDS-PAGE as multiple bands of around 65 KDa [14] and EG2 has been detected as a single band of about 47 KDa [38]. Furthermore, consistent with a previous report [38], the wild-type strain in the current study was observed to secrete little protein into the supernatant (Fig 6, Lane 1). Although the protein bands corresponding to the cellulases could be stained with CBB, it proved difficult to distinguish between the bands for CBH1 and EG2 by means of SDS-PAGE (Fig 6, Lanes 2–4). We therefore chose to compare the amounts of CBH2 and CBH1 + EG2 produced by those strains.

We first compared the amounts of cellulases secreted by the strains expressing all three cellulases and harboring either the *TDH3* promoter and *CYC1* terminator or the L5TR cassette (Fig 6, Lanes 5 and 6, respectively). The HR strain secreted about 1.6-times the amount of CBH2 and about 3.0-times the amount of CBH1 + EG2 compared with the strain harboring the *TDH3* promoter and *CYC1* terminator (Table 3), which is consistent with previous results [26, 38]. The amount of CBH2 secreted by the HR strain was about 0.7-times the amount of cellulase secreted by the strains expressing only one cellulase gene and harboring the L5TR cassette. However, the amount of CBH1 + EG2 (Fig 6, Lanes 2–4 and 6; Table 3) and the accumulation of fluorescent protein in the cytoplasm were comparable between the strain simultaneously expressing two fluorescent proteins and the strains expressing only one fluorescent protein harboring the L5TR cassette [26]. The level of CBH2 secretion was much higher than that of CBH1 + EG2 (Fig 6). We currently have no explanation for this discrepancy.

**Table 3:** Comparison of the amounts and activities of the secreted cellulases.

SDS-PAGE lane number (strain)	2	3	4	5	6 (HR)	7 (3B5)	8 (2D9)	9 (4D4)
Expression system	L5TR			*TDH3pro CYC1t*	L5TR	Combinatorial		
Cellulase expressed	CBH1	CBH2	EG2	CBH1 + CBH2 + EG2				
Relative amount of CBH2	nd	1^a^	nd	0.47 ± 0.06	0.76 ± 0.04	1.51 ± 0.11^e^	1.57 ± 0.16^e^	1.46 ± 0.19^e^
Relative amount of CBH1 + EG2	1^b^	nd	0.50 ± 0.05	0.34 ± 0.11	0.98 ± 0.07	1.68 ± 0.08^f^	1.73 ± 0.18^f^	1.53 ± 0.20^f^
Activity of CBH1 + EG2^c^	3.72 ± 0.09	nd	3.19 ± 0.11	3.62 ± 0.50	7.06 ± 0.33	11.9 ± 0.96	10.9 ± 0.21	12.2 ± 1.02
Relative activity of EG2	nd	nd	1^d^	0.81 ± 0.10	1.01 ± 0.07	1.21 ± 0.13	1.13 ± 0.10	1.11 ± 0.15

nd, not detected

^a^, L5TR-CBH2 strain used as standard

^b^, L5TR-CBH1 strain used as standard

^c^, enzyme assayed with resorufin-cellobiose at room temperature for 30 min (μM resorufin/μl supernatant)

^d^, L5TR-EG2 strain used as standard

^e^, No significant difference between three strains (Welch t-test; P<0.05)

^f^, No significant difference between three strains (Welch t-test; P<0.05)

Measurements were performed in triplicate.

In the three strains selected by means of combinatorial screening, 3B5, 2D9, and 4D4, the amounts of CBH2 and CBH1 + EG2 secreted were comparable but were 2.0- and 1.6-times greater, respectively, than that of the HR strain (Fig 6, Lanes 6–9; Table 3). This result is consistent with the difference in total cellulase activity between these two strains (Fig 5). The differences in the amounts of CBH2 secreted by these three strains may be explained by the number of copies of *CBH2* and type of expression cassette harbored by the strain. That is, strains 3B5 and 2D9 each harbored three copies of *CBH2* and the weak L1TC and middle L5TC expression cassette, respectively, whereas strain 4D4 had two copies of *CBH2* and the strong L5TR expression cassette (Table 2).

### Activities of secreted cellulases

To investigate the differences in the activities of CBH1 and EG2 secreted by the HR strain and the strains selected by means of combinatorial screening, we used a specific substrate for each cellulase. First, we used cellobiose labeled at the reducing terminal with resorufin, a fluorescent substrate, to determine the activity of CBH1 (Table 3, row 3). Unexpectedly, EG2 was observed to digest a substantial proportion of this substrate (Table 3, row 3, columns 2 and 4, [31]). Therefore, the activity of CBH1 presented here is actually the combined activities of CBH1 and EG2. Although no significant differences in CBH1 + EG2 activity were observed among strains 2D9, 3B5, and 4D4, their activities were 1.6-times greater than that of the HR strain, which was consistent with the SDS-PAGE results (Table 3, row 2).

Next, we measured the relative activity of EG2 by using AZCL-HE-cellulose as the substrate. This fluorescence-labeled cellulose was specifically catalyzed by EG2 (Table 3, row 4, columns 2–4). Among the EG2-expressing strains that harbored one copy of *EG2* but different expression cassettes, the activities of EG2 were comparable and directly reflected the intensity of the expression cassette they contained (Table 3, row 4, columns 4–9).

### Conversion of Avicel cellulose to ethanol

**Fig 7:**
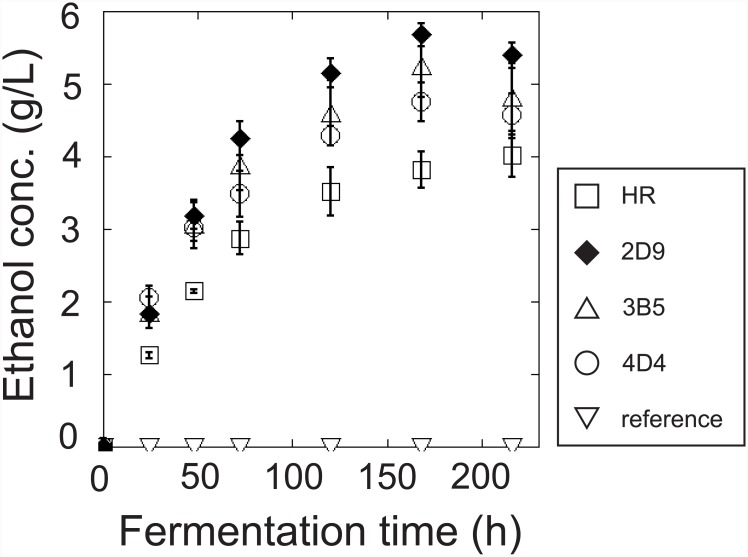
Ethanol fermentation by the transgenic strains obtained from the combinatorial screening. Conversion of Avicel cellulose to ethanol with the external addition of β-glucosidase. The cultures described in Fig 5 legend were used for ethanol fermentation. Symbols are the same as in Fig 5.

The cellulose fermentation activities of the cellulase-expressing strains were investigated by assaying the digestion of partially ordered low-accessibility microcrystalline cellulose (Avicel) (Fig 7). The strains were incubated in YPD medium before being inoculated into buffer solution containing 2% cellulose as the sole carbon source. The strains used in the present study degrade cellulose to a mixture of oligosaccharides such as cellobiose, most of which are not assimilated by the budding yeast. To convert these oligosaccharides to fermentable glucose, β-glucosidase was added to each culture medium. After incubation, the ethanol concentration of the cultures was measured at the indicated times (Fig 7). The three strains obtained from the combinatorial screening (3B5, 2D9, and 4D4) showed greater ethanol production than the HR strain. The ethanol production of the 2D9 strain was significantly higher than those of the 3B5 and 4D4 strains (P<0.05), although all three strains showed similar levels of cellulase activity until 4 h (Fig 5). The ethanol yields of the 2D9, 3B5, and 4D4 strains were 5.7, 5.2, and 4.8 g/l at 168 h, respectively (Fig 7 and Table 2). The level of ethanol production of the 2D9 strain corresponded to approximately 57% of the theoretical maximum yield. This value was twice that of other cellulase-expressing yeasts reported in a previous study [22].

In all five strains with high cellulase activity selected by means of combinatorial screening, single-copy integration of the *EG2* gene was observed, although multiple copies of *CBH1* and *CBH2* were integrated (Table 2). The lack of multiple integrations of EG2 is most likely to have been caused by the low frequency of multiple integrations into the *TRP1* locus, not by the toxicity of EG2 activity. Improving the EG2-integration construct at loci other than at *TRP1* may produce cellulose-digesting strains with higher cellulase activity than those of the strains fabricated in the current study.

Despite the strains obtained in the present study having similar cellulase activities, they exhibited different levels of ethanol production. Furthermore, although the amounts of cellulase secreted were comparable among these strains (Fig 6 and Table 3), the type of expression cassette and copy number of the integrated cellulase genes differed (Table 2). The activities of the cellulases assayed with a cellulase-specific substrate were similar from 30 min to 4 h after incubation (Fig 5 and Table 3). The production levels of ethanol were also similar out to 50 h after incubation (Fig 7). However, at 168 h after incubation, the levels of ethanol production differed significantly among the three strains screened (Fig 7). Although interpretation of these results is difficult, they may be explained by a synergetic effect between the different cellulases [41–43] and by the optimum ratio of cellulase to substrate type and reaction time dynamically changing over time [44].

## Conclusions

In this study, we used combinatorial screening in conjunction with the expression construct library from our previously reported tunable protein-expression system to find the best cellulolytic yeast strain for the production of ethanol. Combinations of four types of cellulase-expression constructs for three different cellulases (CBH1, CBH2, and EG2) were used to successfully construct a pool of yeast transformants with a broad range of cellulase activities. From this transformant pool, the best strains for the hydrolysis of crystalline cellulose were selected. One of these transformants showed a higher ethanol production level (with the external addition of β-glucosidase) than the strains reported in our previous study. Our results indicate that our tunable expression system in conjunction with combinatorial screening is effective for optimizing metabolic flux for the production of target compounds.
